# Transarterial arterial sclerosing embolization for the treatment of propranolol-resistant subglottic hemangioma: Feasibility and effificacy

**DOI:** 10.3389/fonc.2023.1062510

**Published:** 2023-03-02

**Authors:** Zhuang Liu, Dan Song, Liang Wang, Jie Zhou, Changfeng Wang, Jing Li, Jiali Sun, Xian Zhang, Lei Guo

**Affiliations:** Department of Vascular Anomalies and Interventional Radiology, Jinan Children's Hospital, Qilu Children’s Hospital of Shandong University, Jinan, China

**Keywords:** subglottic hemangioma, laryngeal obstruction, feasibility and efficacy, transarterial angiography and embolization, propranolol-resistant infantile hemangiomas

## Abstract

**Purpose:**

To assess the effectiveness and safety of transcatheter arterial sclerosing embolization (TASE) for the treatment of subglottic hemangiomas that did not respond appreciably to propranolol.

**Materials and Methods:**

This study was a retrospective analysis. Of the 101 patients with subglottic hemangiomas admitted to our center, 10 (4 male and 6 female) patients were included in this study. All these patients underwent arterial embolization using Pingyangmycin and polyvinyl alcohol particles (300–500 μm). All patients were examined 1 month after the last treatment and monthly thereafter.

**Results:**

TASE treatment was technically successful in all patients. Ten lesions were located in the subglottic region. The blood supply included the superior thyroid artery, brachiocephalic trunk, facial artery, and ascending pharyngeal arteries. The median maximal diameter of the hemangiomas significantly decreased from 8.5 mm before treatment to 2 mm after TASE (P <.05). The degree of laryngeal obstruction improved in all patients. No serious complications were noted. One patient developed fever postoperatively, and three patients had a mild cough.

**Conclusions:**

For even subglottic hemangiomas with suboptimal efficacy of propranolol, TASE significantly reduced the size of hemangiomas with minimal adverse effects. It had a positive effect on the improvement of airway stenosis caused by subglottic hemangioma with poor effect of oral propranolol.

## Introduction

Infantile hemangiomas (IHS) have a prevalence of 5% and are the most common benign tumors in infants. More than 57% of hemangiomas occur in the maxillofacial region (1). Subglottic hemangioma (SGH) is a rare hemangioma that accounts for only 1.5% of cases with congenital anomalies and may cause severe airway obstruction (2). The mortality rate of untreated SGH is as high as 50%, which is a challenge for clinicians. Subglottic hemangiomas are not easily detected, and diagnosis relies on clinical symptoms and laryngoscopy. The typical finding on laryngoscopy is a pink or blue compressible mass under the glottis, which is usually submucosal, asymmetric, and smooth.

In the past, treatment options for subglottic hemangiomas included steroids (systemic or intralesional), oral propranolol, laser ablation, open resection, and tracheostomy. Propranolol has been shown to be effective in the treatment of hemangiomas and is considered a first-line drug; however, for the treatment of propranolol-resistant infantile hemangiomas (PRIH), especially subglottic hemangiomas, there are few reports in the literature, and a widely recognized safe and effective treatment option is lacking. In our center, transcatheter arterial sclerosing embolization (TASE) has been widely used in the treatment of IHS (3).

This study aimed to test the efficacy and safety of TASE in the treatment of propranolol-resistant subglottic hemangiomas.

## Materials and methods

This retrospective analysis of the data of 101 patients treated for subglottic hemangioma was conducted between August 2015 and June 2022. Ten patients with PRIH received TASE treatment in the Department of Vascular Abnormalities and Interventional Radiology (**Table 1**). All patient guardians consented to the treatment protocol and publication of anonymous data for submission, and the Ethics Committee approved the study protocol.

**Table 1:** Clinical Characteristics of the study patients.

Follow-up (month)	Lesion maximum diameter before TASE(mm)	Lesion maximum diameter after TASE(mm)	Efficacy
14	25	0	excellent
6	0.9	0	excellent
4	12	2	excellent
3	7	0	excellent
2	9	5	general
10	10.4	4	good
8	7	2	good
30	10	0	excellent
3	8	2	good
2	8	2	good

### Patients

Ten infants (4 male and 6 female) diagnosed with subglottic hemangioma and treated with propranolol with unsatisfactory symptom improvement were included in this study (laryngeal obstruction grade not reduced after propranolol treatment for at least 4 weeks, 2 mg/kg/d). The diagnosis of subglottic hemangioma (SGH) is based on medical history; clinical manifestations; and doppler ultrasonography, enhanced CT, and electronic bronchoscopy findings. According to the classification and nomenclature of vascular anomalies proposed by the International Society for the Study of Vascular Anomalies, the exclusion criteria included other vascular malformations and surgical contraindications (4).

The chief complaint of patients with subglottic hemangioma was wheezing or dyspnea, and data on clinical manifestations, imaging examinations, treatment processes, and complications were collected from patient files. The results of electrocardiogram, coagulation factors, liver and kidney function, and routine blood tests were normal for all patients.

### Treatment protocol

The femoral artery approach was used in all operations, and selective arteriography of the bilateral carotid arteries and bilateral subclavian arteries was performed using a 4F Super Cobra catheter (Cook Medical, Bloomington, Indiana) to identify the blood supply arteries of the IH.

After identification of the feeding artery, superselective catheterization of the main feeding artery was performed using a 2.1F microcatheter (Merit Medical System Inc, South Jordan, UT).

Via angiography, polyvinyl alcohol particles (PVA, 300–500 μm) and pingyangmycin, were used to embolize the feeding arteries with the goal that the blood flow velocity in the supply artery was reduced. All major blood supply arteries were embolized, and radiation protection, especially protection of the gonads, was implemented intraoperatively. 

### Therapeutic evaluation standard

All children were evaluated at the clinic 1 month after treatment. Follow-up examinations were conducted once a month, patients underwent enhanced CT or color Doppler ultrasound. Other clinicians evaluated the following treatment effect according to two factors during the 1–3-month follow-up period: improvement of airway compression symptoms, reduction of maximum diameter, or attenuation of blood supply.

Efficacy was classified into four grades considering the reduction in maximum diameter during follow-up: poor (0–25% reduction), general (26–50% reduction), good (51–75% reduction), and excellent (76–100% reduction).

Evaluation of improvement in clinical symptoms was primarily based on the degree of laryngeal obstruction. Grade IV: extreme dyspnea, three concave signs, and decreased consciousness and incontinence; Grade III: marked dyspnea, tachypnea, marked stridor and a depressed inspiratory thoracic cage, restlessness, and increased heart rate; Grade II: breathlessness was noted when calm, and when active, it significantly worsened, accompanied by stridor; Grade I: there was no significant dyspnea when calm, an inspiratory breathlessness developed only during activity or when crying, and stridor sounds and possible thoracic invaginations during strenuous exercise.

Patients were assessed for possible adverse events during postoperative and follow-up visits. Complications were classified according to the Society of Interventional Radiology guidelines. Minor complications were defined as those that exhibited clinical symptoms that were not significant and did not require treatment, such as a mild fever. Major complications were defined as those that required an increased level of care, surgery, hospitalization, or permanent adverse outcomes.

All analyses were performed using Statistical Package for the Social Sciences (version 17; IBM Corporation, Chicago, Illinois, USA). The t-test was used for univariate analysis of continuous data. Statistical significance was defined as P <.05.

## Results

### Angiographic manifestations

In the present study, the feeding artery of the hemangioma mostly originated from the branch of the inferior thyroid artery or the branch of the thyrocervical trunk in all patients. Among the 10 patients, the superior thyroid artery was involved in supplying blood to 9 patients, the thyrocervical trunk was involved in supplying blood to 3 patients, and the ascending pharyngeal artery was involved in supplying blood to 1 patient. In contrast, the nidus and refluxing veins were clear, but the vein was no earlier than normal. PVA particles with diameters of 300–500 μm were selected for all patients according to Mulliken’s classification (5).

### Therapeutic evaluation

**Figure 1:**
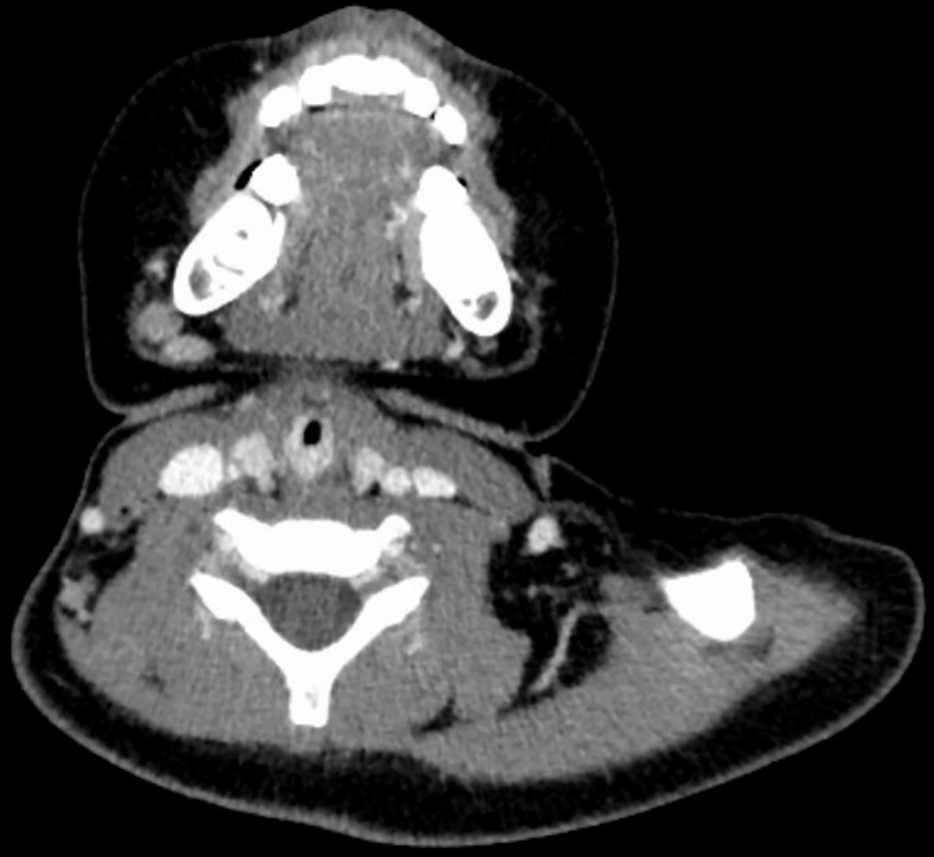
CT image of No. 8 patient before embolization treatment, with marked enhancement of hemangioma and airway stenosis.

**Figure 2:**
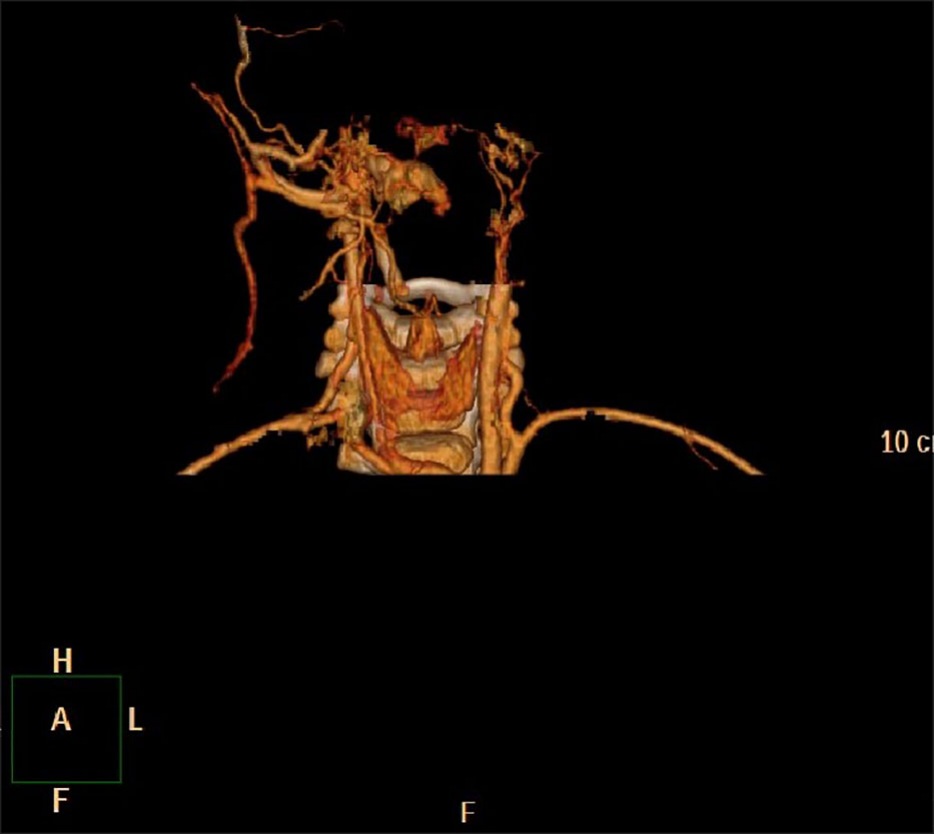
No. 8 patient 3D reconstruction of subacoustic hemangioma before treatment.

**Figure 3:**
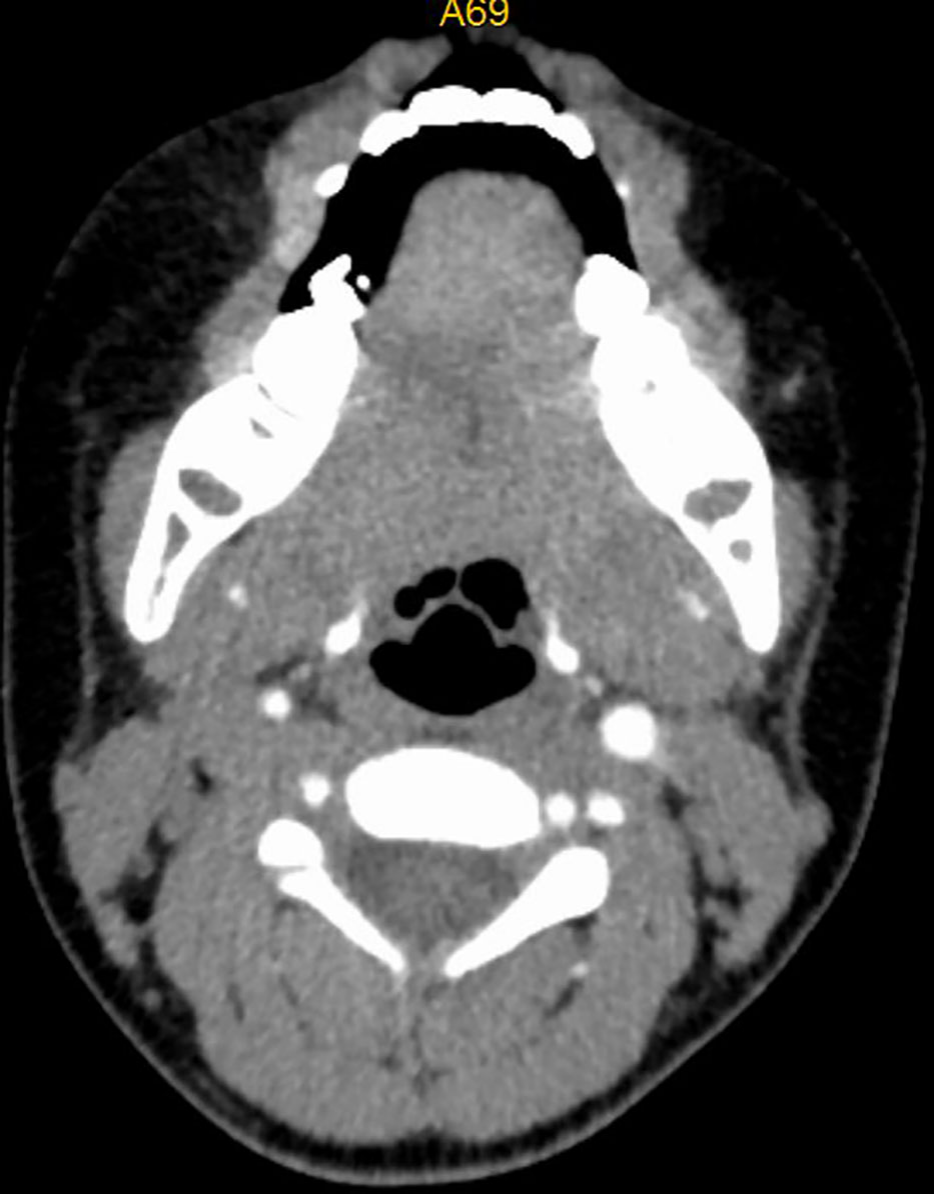
CT image of No. 8 patient after embolization treatment, hemangioma disappeared, no airway stenosis.

**Figure 4:**
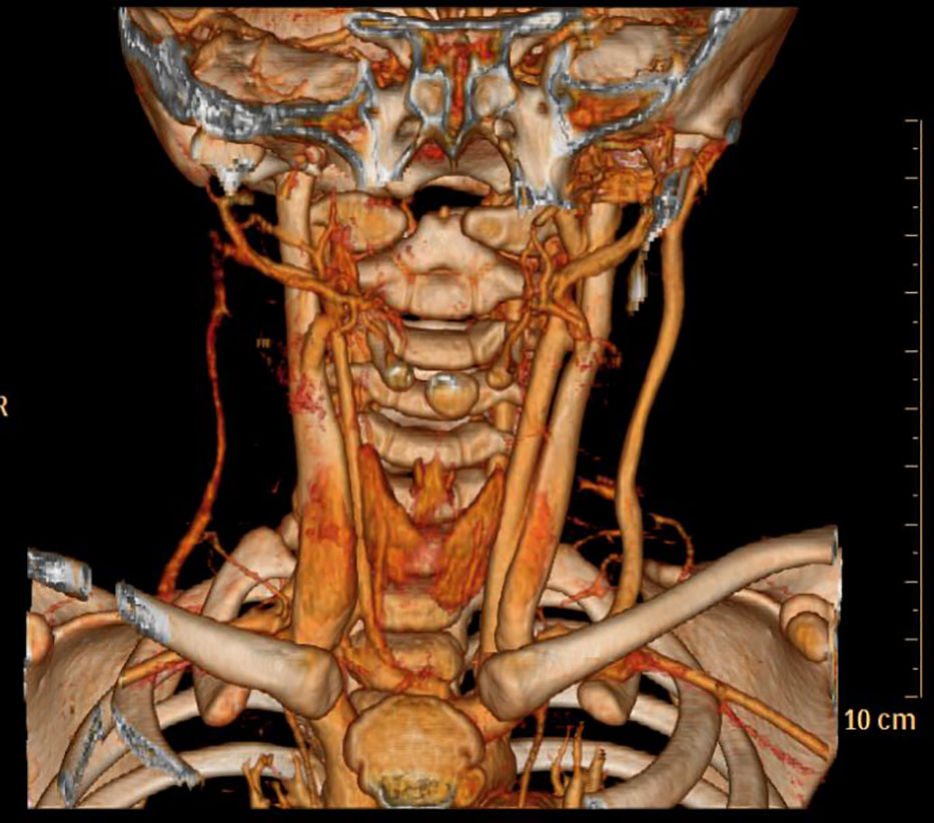
No. 8 patient Post-treatment 3D reconstruction,the hemangioma has disappeared.

All cases of SGHs were successfully treated. The median maximum diameter of the lesion was reduced from 8.5 mm before treatment to 2 mm(P<.05). The degree of laryngeal obstruction improved, with an overall response rate of 100.0% (**Figures 1**–**4**).

### Complications

There was one case of fever and three cases of cough, which resolved spontaneously within 2 days, and no serious complications occurred in any patient.

## Discussion

Infantile hemangiomas are common benign tumors with abnormal vascular endothelial cell proliferation and are characterized by rapid growth of endothelial cells at birth, followed by a slow degenerative period lasting for years, which may lead to complete disappearance of the tumor (6).

Infantile SGHs are potentially life-threatening hemangiomas. Although hemangiomas usually begin to resolve spontaneously on their own after 18–24 months, they are likely to cause airway obstruction because they compress the airway.

These patients typically present with signs or symptoms, such as cyanosis, hoarseness, stridor, barking cough, wheezing, chest and abdominal retching, and respiratory distress. Because of the electronic bronchoscopy and CT imaging techniques, laryngeal examination can help us detect subglottic lesions early (7).

Owing to the dangerous nature of SGHs, observation is not optimal in the clinic.Pharmacological or surgical treatment of SGHs is more commonly used. Oral propranolol has been suggested as the first-line therapy for treating SGHs (8). If propranolol is not effective in the treatment of hemangioma, glucocorticoid is another option. However, there are significant adverse effects associated with the use of glucocorticoids (9).

The propranolol-resistant phenomenon is not very rare.In Tyler Schwartz’s 2017 study, the 49 patients with subglottic hemangioma were treated with oral medication and 6 patients failed treatment (10). In a 2020 study by Lei Guo, 21 patients all had propranolol-resistant hemangioma (2). Zhaobo Liu (11) and J GOSWAMY (12) also reported the failure of oral propranolol in the treatment of subacoustic hemangioma.

Y BAJAJ recommends a propranolol dose of 2 mg/kg/d for subacoustic hemangioma (13), while Scott Hardison believes that propranolol doses up to 3 mg/kg/d are more effective in the treatment of hemangioma (14). According to the existing expert consensus on the treatment of hemangioma in our country, the dose of propranolol for the treatment of hemangioma does not exceed 2 mg/kg/d (15). Therefore, we use propranolol at a dose of 2 mg/kg/d for the treatment of subacoustic hemangiomas.

Sirolimus is considered an effective drug for the treatment of hemangioma by inhibiting the mTOR pathway (16), and a few cases of sirolimus in the treatment of hemangiomas have been infrequently reported (16, 17). However, studies on the safety of sirolimus in pediatric patients are lacking.

A recent large randomized controlled trial revealed that atenolol has similar efficacy and fewer adverse events in the treatment of infants with problematic IHs when compared with propranolol. Oral atenolol can be used as an alternative treatment option for IH patients requiring systemic therapy (18). Another randomized controlled trial also showed that oral nadolol was noninferior to oral propranolol, indicating it may be an efficacious and safe alternative in cases of propranolol unresponsiveness or adverse events, or when faster involution is required (19).

In addition to oral pharmacotherapy, other treatment modalities also have significant disadvantages. Infants with tracheostomies have high mortality and morbidity rates. Steroid injection plus intubation prolongs the stay in the intensive care unit. Laser ablation can lead to scarring and acquired strictures, surgical excision is more traumatic (13), and tracheostomy affects child growth and development (20).

Transcatheter arterial embolization has been shown to be effective in the treatment of hemangioma (3). In our 10 patients treated with TASE, the diameter of the hemangioma was reduced, and the degree of laryngeal obstruction was reduced in all patients. Patients experienced minor complications; however, one patient’s high temperature returned to normal temperature after physical cooling, and three patients’ cough, probably due to stimulation of the local mucosa by the laryngeal mask during anesthesia, resolved after being aerosolized.

## Conclusion

Our study suggests that TASE is a safe, effective, and promising treatment option to rapidly alleviate laryngeal obstruction caused by propranolol-resistant subglottic hemangioma.

## Data availability statement

The original contributions presented in the study are included in the article/supplementary material. Further inquiries can be directed to the corresponding author.

## Ethics statement

The studies involving human participants were reviewed and approved by Children’s Hospital Affiliated to Shandong University. Written informed consent was obtained from the individual(s), and minor(s)’ legal guardian/next of kin, for the publication of any potentially identifiable images or data included in this article.

## Author contributions

ZL Collect case data and formulate diagnosis and treatment plan, Writing - Review & Editing. DS: Doctor patient communication and perioperative management. JS: Data analysis. XZ: Data analysis. JZ: Implement treatment plan. CW: Perioperative management. LW: Implement treatment plan. LG: Formulate inclusion and exclusion criteria, Writing - Review & Editing. All authors contributed to the article and approved the submitted version.
